# Role, Laboratory Assessment and Clinical Relevance of Fibrin, Factor XIII and Endogenous Fibrinolysis in Arterial and Venous Thrombosis

**DOI:** 10.3390/ijms22031472

**Published:** 2021-02-02

**Authors:** Vassilios P. Memtsas, Deepa R. J. Arachchillage, Diana A. Gorog

**Affiliations:** 1 Cardiology Department, East and North Hertfordshire NHS Trust, Stevenage, Hertfordshire SG1 4AB, UK; vassilios.memtsas@nhs.net; 2 Centre for Haematology, Department of Immunology and Inflammation, Imperial College London, London SW7 2AZ, UK; d.arachchillage@imperial.ac.uk; 3 Department of Haematology, Imperial College Healthcare NHS Trust, London W2 1NY, UK; 4 Department of Haematology, Royal Brompton Hospital, London SW3 6NP, UK; 5 School of Life and Medical Sciences, Postgraduate Medical School, University of Hertfordshire, Hertfordshire AL10 9AB, UK; 6 Faculty of Medicine, National Heart and Lung Institute, Imperial College, London SW3 6LY, UK

**Keywords:** factor XIII, fibrin, endogenous fibrinolysis, thrombosis, coagulation

## Abstract

Diseases such as myocardial infarction, ischaemic stroke, peripheral vascular disease and venous thromboembolism are major contributors to morbidity and mortality. Procoagulant, anticoagulant and fibrinolytic pathways are finely regulated in healthy individuals and dysregulated procoagulant, anticoagulant and fibrinolytic pathways lead to arterial and venous thrombosis. In this review article, we discuss the (patho)physiological role and laboratory assessment of fibrin, factor XIII and endogenous fibrinolysis, which are key players in the terminal phase of the coagulation cascade and fibrinolysis. Finally, we present the most up-to-date evidence for their involvement in various disease states and assessment of cardiovascular risk.

## 1. Introduction

Arterial thrombosis, especially in the context of myocardial infarction (MI), is most commonly attributable to atheromatous plaque disruption and contact of procoagulant substrate (subendothelial cells, tissue factor, collagen) with blood, leading to platelet activation, aggregation and formation of an occlusive platelet-rich clot [1]. On the other hand, venous thrombosis is most likely initiated by the interaction between dysfunctional—but intact—endothelium expressing cell adhesion molecules and plasma hypercoagulability combined with reduced blood flow, which subsequently leads to the formation of a red blood cell (RBC)-rich thrombus [2]. Fibrin clot formation is the ultimate common event in the coagulation cascade leading to thrombus formation, and there is emerging evidence that the structure and properties of these clots may determine the outcome and persistence of the thrombus [3]. Endogenous fibrinolysis is an important physiological countermeasure against development and lasting arterial or venous thrombosis, and the dysfunctional fibrinolytic process leads to both acute thrombosis and a chronic thromboembolic disease process. Furthermore, the clot-stabilising effect of factor XIII (FXIII), which is found in cells and plasma, is thought to contribute to pathological thrombosis. In this review, we present the (patho)physiological role of fibrin and FXIII, which play a pivotal role in the terminal phase of the coagulation cascade as well as the role of impaired endogenous fibrinolysis in pathological thrombus formation. Finally, we summarize the currently available evidence for the involvement of these in arterial and venous thrombotic events in various diseases as well as their value in predicting future cardiovascular risk.

### 1.1. Physiological Role of Fibrin

Fibrinogen is the product of three genes clustered on chromosome 4 [4], namely FGA, FGB and FGG, each of which specifies the primary structure of one of its 3 polypeptide chains Aa, Bβ and γ, respectively [5,6]. Fibrinogen is primarily synthesized in the liver at a rate of 1.7–5 g/day [7]; however, its expression is strongly upregulated in response to inflammation [6]. Approximately three-quarters of fibrinogen in humans is present in the plasma, at concentrations between 1.5–3.0 mg/mL, but it can also be found in platelets, lymph and interstitial fluid [8].

Fibrinogen is converted to fibrin during the final stages of the coagulation cascade. The trigger for this conversion is the thrombin-mediated catalytic cleavage of fibrinogen into fibrin. Thrombin cleaves N-terminal fibrinopeptides from the Aa- and Bβ-chains and produces fibrin monomers that further polymerize into fibrin fibers [9]. This results in a drastic change in solubility, which causes the polymerization, aggregation and branching of molecules and, ultimately, the formation of insoluble fibrin fibers [3], which give rise to the fibrin meshwork that is essential for the mechanical stability of a newly formed clot [10,11,12,13,14]. To further stabilize the clot against proteolytic and mechanical insults, the emerging fibrin fibers are covalently crosslinked by the activated form of factor XIII (FXIIIa), a plasma transglutaminase, which is activated by thrombin in the presence of calcium (Ca^2+^) [8]. Before cross-linking, the fibrin polymerization is reversible [15]; however, after cross-linking, polymerization becomes irreversible. This ultimately leads to the formation of a rigid but simultaneously elastic structure that is capable of withstanding mechanical stress and is less susceptible to proteolytic disruption [3]. From a purely mechanistic point of view, numerous studies [16,17] have shown that the fibrin network structure in a clot is an important determinant of the viscoelastic properties of the clot and can significantly influence its susceptibility to lysis. All of these parameters are heavily dependent on the kinetics of fibrin polymerization. Thrombin concentration at the time of gelation has a profound effect on fibrin clot structure, an effect previously demonstrated in vitro [18]. Clots formed at low thrombin concentrations are coarse and are composed of thick fibrin fibers, which are highly susceptible to fibrinolysis [19]. On the other hand, the presence of high thrombin concentration leads to formation of dense clots with thin fibers that are highly branched and relatively resistant to fibrinolysis [18,19,20,21,22].

A recent proteomic study [23] has identified at least 48 proteins that are incorporated by cross-linking into the growing plasma clot by FXIII. For the purposes of this review, the most important ones appear to be a_2_-antiplasmin (A2AP) [24], plasminogen activator inhibitor-1 (PAI-1) [24], plasminogen activator inhibitor-2 (PAI-2) [25,26], thrombin activatable fibrinolysis inhibitor (TAFI) and complement C3 [27], as they act as regulators of fibrinolytic activity (see the relevant section below). Furthermore, clot quality is influenced by cells and cell-derived components that are present at the site of injury, such as RBCs, neutrophils and neutrophil extracellular traps [28,29,30,31,32,33,34,35].

Once a clot is formed, the fibrinolytic system is also activated, a process predominantly regulated by the tissue plasminogen activator (t-PA)-mediated conversion of plasminogen to plasmin. Fibrin serves as both a co-activator and a substrate for plasmin, which is physiologically relevant, as it restricts fibrinolytic activity to the location of the formed clot. This is a crucial step in achieving a dynamic equilibrium between clot formation and lysis and to prevent uncontrolled clot propagation [3]. The effectiveness of the fibrinolytic process depends both on the regulation of fibrinolytic enzyme activity, as well as the physical characteristics of the fibrin network itself, such as fiber diameter, pore size, and extent of branching. Weisel et al. [36] have demonstrated that, in general, clots composed of thicker fibers appear to lyse faster compared to clots that are made up of thinner fibers. Finally, platelet aggregation, clot retraction [37] and clot stretching [38], amongst others, affect the rate of clot lysis. 

### 1.2. Assessment of Fibrin Clot Structure and Permeability

Since the properties of the fibrin fiber network in a clot can determine its mechanical stability and resistance to the endogenous fibrinolysis, methods of assessment of the clot structure have been developed to assess cardiovascular risk. Permeability of the fibrin clot is a measure of how tightly packed the fibrin clot is. This can be assessed in citrated plasma, mixed with thrombin to enable fibrin clot formation. The volume of buffer flowing through the gel formed is subsequently used to calculate the clot permeation coefficient [39]. The structure and properties of fibrin networks in clots can be further characterized using scanning confocal microscopy and electron microscopy to determine parameters such as fiber diameter, fiber length, fiber density, number of branching points and the size of pores present in the mesh [8].

### 1.3. Involvement of Fibrin Clot Structure in Disease States

The association between fibrin clot structural characteristics and clinical pathologies suggests that the fibrin network structure is indeed a critical determinant of hemostasis and thrombosis. Numerous studies have demonstrated that dense networks of thinner and more compact fibrin fibers are associated with increased thrombotic risk [40,41,42], whereas more coarse networks comprising thicker and less compacted fibers are linked to increased risk of bleeding and are more susceptible to fibrinolysis [18,22,40,41,42,43,44]. Growing evidence suggests the idea that abnormal fibrin clot characteristics (such as permeation, turbidity, compaction and lysis assays) may represent a novel risk factor for arterial and venous thromboembolism (VTE) [45].

There are two recognised types of inherited fibrinogen disorders. Type I (which refers to reduced quantity of fibrinogen) includes afibrinogenemia (plasma levels < 0.1 g/L) and hypofibrinogenemia (plasma levels 0.1–1.5 g/L). Type II (which refers to a qualitative fibrinogen abnormality), also known as dysfibrinogenemia, describes a state of normal fibrinogen levels with low functional activity [46]. Interestingly, inherited fibrinogen disorders confer both an increased bleeding risk and an increased risk of thromboembolic complications. 

Altered clot structure in the context of disease was first observed in individuals with advanced coronary artery disease (CAD) [47]. Dense fibrin fiber networks, which were ~30% less permeable, were demonstrated in young (<45 years) male subjects [47,48]. Furthermore, patients aged < 50 years after a first MI had longer clot lysis, which was associated with increased body mass index, blood pressure and C reactive protein [49]. Similar but milder alterations in fibrin structure were reported by Mills et al. [41] in first-degree healthy relatives of patients with premature CAD, suggesting a possible inherited predisposition to altered fibrin network structure and disease. Collet et al. [50] demonstrated that fibrin clot architecture was similarly altered in young survivors of MI, exhibiting increased stiffness, shorter fibrin fibers and hypofibrinolysis. These findings were extended to older individuals with advanced CAD, who had increased fiber density and resistance to fibrinolysis compared to age-matched controls [51]. Reduced clot permeability, lysis time (LT) and turbidity, attributed to more tightly packed and less porous fibrin networks, have also been associated with significant complications of CAD, namely stent thrombosis and no-reflow phenomenon [52]. Finally, reduced fibrin clot permeability and susceptibility to lysis has also been observed in patients with a history of the no-reflow phenomenon after acute MI [53].

Similar abnormal fibrin clot properties to those encountered in acute MI patients have also been documented in patients during the acute phase of ischaemic stroke [54,55], suggesting that reduced clot permeability and hypofibrinolysis contribute to the mechanism of thrombosis. More importantly, in a study performed by Rooth et al. [56], the changes observed during the acute phase of ischaemic stroke persist beyond 60 days from the index event, suggesting that impaired fibrinolysis is a persistent characteristic in these patients. Individuals with acute ischaemic stroke and concomitant CAD exhibited prolonged clot lysis compared to controls without CAD history. Fibrin clot compaction was correlated with a neurological deficit on both admission and discharge of subjects with acute ischaemic stroke [55]. More compact plasma clots (and thus more resistant to fibrinolysis) were generated from plasma samples of individuals with cryptogenic stroke between 3 and 9 months earlier [57]. It should, however, be noted that ischaemic stroke is a rather heterogeneous pathology, and it is not clear whether all types of ischaemic strokes share the same underlying fibrin network properties. 

Peripheral arterial disease (PAD) is another vascular condition where denser fibrin clots with reduced permeability and susceptibility to lysis have been observed [47,58]. Worse clinical outcomes, namely increased risk of thromboembolism and further progression of PAD, were associated with the adverse clot phenotype. Altered fibrin clot structure, in the form of poor permeability, increased rigidity/fiber thickness and resistance to fibrinolysis was observed in 34 young individuals with intermittent claudication due to PAD [59]. Hypofibrinolysis was also identified as a risk factor (2.3-fold increase in OR) for arterial thrombosis at a young age in a study by Guimaraes et al. [60]. Additionally, altered fibrin clot architecture and function was observed in healthy first-degree relatives of PAD patients [61]. Patients with PAD, abdominal aortic aneurysm and end-stage renal failure have been shown to have increased fibrinogen levels [62,63,64]. It is, however, noteworthy that fibrinogen levels appear to be of limited value in individualised risk assessment of these patients [62]. It is unclear whether the observed fibrin clot abnormalities are a biomarker of an underlying pathophysiological mechanism or a causative in the disease etiology [65]. 

With regards to VTE, reduced fibrinolytic potential has been demonstrated in patients following their first deep vein thrombosis (DVT) episode [66] and is a predictor of recurrent VTE including pulmonary embolism [67]. In those patients, clot LT above the 90th percentile was associated with a 2-fold increase in DVT risk. Idiopathic VTE patients, as well as their asymptomatic first-degree relatives, were found to have lower clot permeability, lower compaction and prolonged clot LT compared to controls, with those changes being more pronounced in patient subjects. Interestingly, fibrin clot samples obtained from pulmonary embolism patients were more permeable, more susceptible to lysis and less compact compared to those of DVT patients [40]. It is unclear whether VTE patients with risk factors such as trauma, surgery, known thrombophilia or cancer exhibit the same fibrin clot property characteristics, as these were excluded from the above study.

Another group of patients in whom reduced fibrin clot permeability and resistance to fibrinolysis has been documented is those with end-stage renal disease [68,69]. Purified fibrinogen samples obtained from individuals on chronic haemodialysis showed evidence of glycosylation and guanidinylation. Compared to healthy controls, fibrin clots with the above biochemical alternations have been shown to have significantly thinner, more compact and denser fibrin fiber networks [70]. Furthermore, the presence of denser fibrin networks was independently associated with higher mortality. During a 3-year follow-up, clots made from baseline plasma obtained from long-term hemodialysis patients who died of cardiovascular causes were significantly less permeable and less susceptible to lysis, compared to clots formed from controls without cardiovascular disease, indicating a possible link between altered fibrin properties and worse outcome patients with renal failure [71].

Prospective studies are needed to further elucidate whether fibrin clot parameters can reliably predict individuals at an increased risk of thromboembolic events (arterial or venous), both in the general population as well as in subjects with established disease. 

### 1.4. Physiological Role of FXIII

FXIII is involved in the final part of the common coagulation pathway. Its discovery can be traced back to 1948 when Laki and Lorand [72] first identified a serum factor that made fibrin clots insoluble in concentrated urea solution, resulting in the term “protein-fibrin-stabilizing factor”. It was later purified in 1961 by Lowey et al., who also reported its enzymatic activity. In the same year, Duckert et al. [73] realised its clinical importance when they published on a pediatric patient with severe bleeding diathesis, impaired wound healing and abnormal scar formation, in what probably appears to be the first documented case of FXIII deficiency.

FXIII is a member of the transglutaminase family and is found in both cellular and plasma fractions. It consists of subunits A (catalytic) and B (non-catalytic). The A subunit consists of a sandwich domain, a catalytic domain, an activation peptide (AP) and two barrel domains. The B subunit’s main defining molecular characteristic is the repetitive ten sushi domain. The cellular form of FXIII is found in multiple cell types, including megakaryocytes, platelets (alpha granules) [74], monocytes and osteoblasts [9]. The cellular form consists of two identical FXIII-A subunits, which exist as a homodimer. Most platelet FXIII is derived from megakaryocytes during platelet production, but platelets may also uptake a small fraction of their FXIII from plasma [9], as well as translate FXIII messenger ribonucleic acid de novo [75]. The plasma form exists as a heterotetramer (FXIII-A_2_B_2_) composed of two FXIII-A and 2 FXIII-B subunits and circulates in complex with fibrinogen [76]. Both plasma and platelet FXIII forms are believed to contribute to blood coagulation via means of fibrin clot stabilisation. During its activation, thrombin catalyzes the cleavage of the AP from the FXIII-A subunit [77,78]. Subsequently, (Ca^2+^) promotes the dissociation of the A and B subunits, and the FXIII-A becomes an active transglutaminase mediating the fibrin cross-linking and clot stabilisation [79]. FXIII binding to fibrin(ogen) facilitates the dissociation of the B subunits [80,81,82], essentially ascribing fibrin(ogen) with an important regulatory role in FXIII activation. Finally, it should be noted that cellular FXIII can also be activated without cleavage of the AP. Cellular FXIII can be proteolytically activated by thrombin and the intracellular (Ca^2+^)-sensitive protease calpain in vitro [83]. When, however, FXIII activation is mediated by AP cleavage, the AP is released into circulation [78,84] and has been used as a biomarker of acute ischaemic stroke [85].

Once activated, FXIII facilitates the cross-linking of fibrin fibers, stabilizing the insoluble clot against dislodgement by high shear stress [86]. Rather interestingly, this cross-linking has only a minor effect (~12%) on fibrin network density [20,87,88,89]. However, it substantially alters the structure of individual fibrin fibers by promoting protofibril coupling within the fiber itself [90], which causes fiber compaction. This has two main effects: firstly, it significantly increases the elastic modulus of individual fibers [91,92,93] (fiber stiffening), as well as the whole clot network [94], making fibers more resistant to deformation under low strain [90]. Secondly, it decreases the size of the pores within individual fibers, with potentially profound effects on the ability of fibrinolytic molecules (such as t-PA), to diffuse within the clot [9]. 

**Figure 1:**
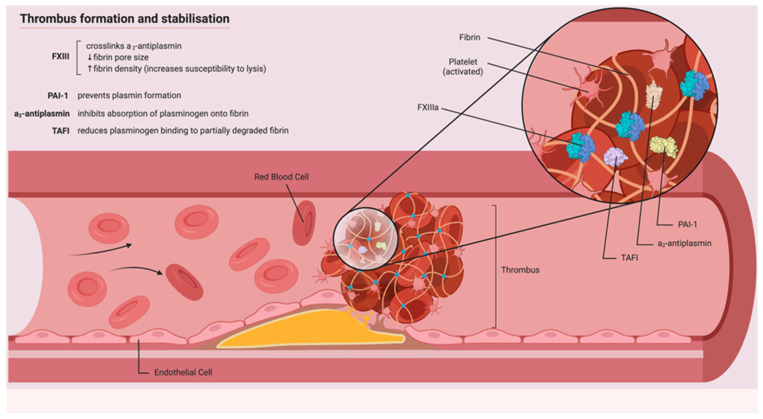
Thrombus formation and stabilisation: Coagulation factor XIII (FXIII) crosslinks fibrin fibers and stabilises the insoluble clot against dislodgement by high shear stress. It also decreases the size of the fibrin pores, reducing the ability of fibrinolytic molecules (such as tissue plasminogen activator (t-PA)) to diffuse within the clot. In addition, FXIII mediates the crosslinking of various antifibrinolytic proteins within the fibrin clot structure itself, further inhibiting clot dissolution. Major regulators of fibrinolysis include a2-antiplasmin (A2AP), thrombin-activatable fibrinolysis inhibitor (TAFI) and plasminogen activator inhibitor (PAI-1).

Crucially, beyond the fibrin fiber cross-linking described above, at least some of the anti-fibrinolytic action of FXIII is mediated by its ability to crosslink antifibrinolytic proteins within the fibrin clot itself (Figure 1). These include A2AP [95,96], TAFI [97] and PAI-1 [98]. In particular, cross-linked A2AP, which is essential for the inhibition of fibrinolysis, remains fully active and protects the formed clot from spontaneous fibrinolysis by t-PA-induced plasminogen activation on the surface of fibrin fibers [99]. 

Finally, there are data suggesting that FXIII-mediated cross-linking also has a significant effect on thrombus RBC content. Wolberg et al. [100] have demonstrated that FXIIIa promotes red blood cell retention in contracting clots by crosslinking fibrin a-chains. Similarly, Aleman et al. [101] have observed reduced RBC retention in contracted human whole blood clots, in the presence of FXIII inhibitors or reduced concentration of FXIII. Since clot contraction packs and deforms RBC within the clot, it has the potential to decrease permeability [30], rendering it a major determinant of clot composition.

Given the above, it is clear that FXIII exerts a multitude of effects on clot structure and function. These are not limited solely to the simplistic mechanical cross-linking of individual fibrin fibers within the clot network but extend beyond this, affecting the biochemical and cellular composition of the clot itself, conferring additional resistance to fibrinolysis. There is growing evidence from epidemiological, biochemical and animal model studies suggesting that FXIII is indeed an important determinant of thrombus composition and stability [9].

Beyond its critical role in the coagulation cascade, other numerous “novel” functions of FXIII (especially of its cellular form) have been identified and are under investigation. It plays a role in extracellular matrix deposition and osteoblast differentiation, with inhibition of FXIIIa resulting in reduced fibronectin and collagen matrix assembly and decreased bone mineralization [102]. Furthermore, FXIII has been reported to be present in adipocytes, where it acts as an antagonist for adipocyte differentiation and lipid accumulation [103]. In addition, FXIIII has been identified as having an important function in wound healing and tissue repair by enabling cross-linking of proteins in the extracellular matrix and promoting cellular signaling between leukocytes and endothelial cells, which enhances cell motility, proliferation, angiogenesis and contributes to immune processes [104,105]. Interaction with the extracellular matrix probably also explains why FXIII is essential for maintaining pregnancy [74], as evidenced by the fact that women with FXIII deficiency suffer from recurrent spontaneous abortions early in the course of pregnancy [106]. Since these functions of FXIIII are beyond the scope of this review article, the reader is encouraged to refer to the excellent articles above for further details.

## 2. Measurement of FXIII

Unlike other coagulation factors, factor XIII level does not influence the routine coagulation screen tests such as prothrombin time or activated partial thromboplastin time. Therefore, high clinical suspicion and prompt investigations with specific tests to detect factor XIII level are required in clinical practice. There are several available assays for performing quantitative and qualitative assessment of FXIII levels and activity, including testing for the presence of FXIII inhibitors. These can broadly be categorised as follows.

### 2.1. FXIII Activity Assays

#### 2.1.1. Clot Solubility Test

This assay evaluates the stability of cross-linked fibrin. Clotting of citrated plasma is initiated by the addition of (Ca^2+^) and/or thrombin. It is then exposed to a protein denaturating agent (such as urea, acetic acid or momoacetic acid). The presence of cross-linked fibrin can be detected as it is more stable (and thus more resistant to denaturation), compared to un-crosslinked fibrin, which readily denatures and dissolves into solution. This is a simple, cost-effective and simple-to-implement method. It is, however, able to detect only severe FXIII deficiency.

#### 2.1.2. Quantitative Activity Assays

These assays rely upon the two different activities of FXIII: The transglutaminase activity and the isopeptidase activity. In short, the transglutaminase activity leads to the release of an ammonia molecule. The isopeptidase activity is characterised by the hydrolysis of isopeptide bonds.

I. Ammonia assays (the most commonly used in clinical laboratories) quantify the ammonia released due to FXIII’s transglutaminase activity, utilising spectrophotometry. This acts as an indirect measurement of transglutaminase activity and is used to quantify overall FXIII activity.
II. Amine-incorporation assays also quantify FXIII’s transglutaminase activity by assessing the isoamide bond formation, catalysed by FXIIIa. A labeled amine substrate, such as 5-biotinamidopentylamine, is paired with a glutamine-containing protein, such as casein. The activity of FXIIIa is quantified by measuring the residual unincorporated labeled amine. Importantly, amine incorporation assays may yield falsely elevated FXIII activity levels in subjects with the common Val34Leu polymorphism, which is present in ~25% of European Caucasians [107]. Thus, it is rarely used in the clinical setting.
III. Isopeptidase assays measure the FXIIIa-dependent hydrolysis of γ:ε isopeptide bonds. A FXIII substrate (A101) with a fluorophore and a fluorescent quencher linked by γ:ε isopeptide bonds is formulated. The isopeptidase-dependent release of the quencher unmasks A101 fluorescence which is detected by a spectrofluorometer, allowing for dynamic measurement of FXIII enzyme activity.

## 3. FXIII Quantitative Antigen Assays

These assays measure FXIII-A_2_, FXIII-B_2_ and FXIII-A_2_ B_2_ complexes by latex antigen immunoassay. They are used for the detection and classification of FXIII deficiencies, as well as monitoring of response to therapy and are widely used in the UK (~40% of centers). In particular, the HemosIL FXIII-A_2_ assay is approved for use by the United States Food and Drug Administration.

## 4. FXIII Genotyping

The encoding genes of FXIII-A (F13A1) and FXIII-B (F13B) have been mapped in the human genome and are located in positions 6p24–25 and 1q31–32.1, respectively. More than 153 mutations have so far been described. Over 95% of FXIII deficiency cases are due to FXIII-A deficiency [108,109]. The large number of mutations and polymorphisms of the FXIII A and B genes that have been described are comprehensively presented by Biswas et al. [110].

A more detailed overview of the laboratory assessment of FXIII and the standard algorithm endorsed by the Scientific and Standardization Committee of the International Society of Thrombosis and Haemostasis for the diagnosis and classification of FXIII deficiency has previously been reviewed in detail by Dorgalaleh et al. [111] and Durda et al. [86].

### 4.1. Involvement of FXIII in Disease States

Congenital FXIII deficiency is a rare bleeding disorder that is inherited in an autosomal recessive pattern. It usually presents in the neonatal period and can result in severe bleeding, most commonly manifesting in umbilical cord bleeding (80%) [112] or intracranial hemorrhage (30%) in the severe form of the disease. Post-traumatic intracranial hemorrhage is often the first sign of mild to moderate FXIII deficiency in older children [113]. Other presentations include deep and superficial hematomas and prolonged bleeding after trauma or surgery in this age group [114]. Women with congenital FXIII deficiency suffer significant bleeding complications. Menorrhagia is a common symptom. Pregnancies in women with FXIII deficiency have a significant risk of miscarriage, placental abruption and post-partum hemorrhage if not on prophylaxis treatment [115].

Contrary to the common manifestations of bleeding, FXIII has been implicated as a contributing factor to arterial and venous thrombosis. Numerous studies have examined the contribution of FXIII in arterial and venous thrombosis [116,117,118] mostly involving the Val34Leu polymorphism, which causes accelerated release of the FXIII AP and results in earlier activation and thus faster fibrin cross-linking in vitro [119,120].

Meta-analysis of early studies performed to evaluate the effect of Val34Leu polymorphism suggests that the presence of the Val34Leu polymorphism offers modest protection against CAD (odds ratio (OR) 0.81, 95% confidence interval (CI) 0.70–0.92) and VTE (OR 0.85, 95% CI 0.77–0.95) [121].

In a large prospective study (EPIC-Norfolk) evaluating the risk of CAD in association with the Val34Leu variant and fibrinogen levels, it was found that Val34Leu homozygotes in the lowest tertile of fibrinogen concentration had increased risk of CAD, whereas individuals in the highest tertile showed a trend towards reduced risk of CAD [122]. Similarly, in a high-risk Hungarian population, the presence of the Val34Leu allele in those with elevated fibrinogen levels was associated with protection against MI [123]. The presence of the allele, however, did not appear to confer any MI risk reduction in the general population. The proposed mechanism for the effects observed is that the highly active Val34Leu variant of FXIII in the presence of high fibrinogen concentrations gives rise to clots with thicker fibers, which have increased permeability and thus are more susceptible to fibrinolysis [124].

A meta-analysis of 36 studies, performed by Jung et al. in 2017 [125] found that FXIII Val34Leu polymorphism was associated with CAD risk, especially MI, but not with CAD without MI. Of note, the age and sex of the individuals did not appear to affect the relationship between the presence of the polymorphism and CAD risk. In a study of 113 patients [126] who planned to undergo elective coronary artery bypass grafting, the FXIII Val34Leu allele was associated with decreased fibrin clot permeability and efficiency of lysis ex vivo. In 474 patients with DVT enrolled in the Leiden Thrombophilia Study, Val34Leu homozygosity in the context of high fibrinogen levels was found to confer vascular protection to both sexes, especially in those individuals over 45 years of age (OR 0.4, 95% CI 0.2–1.0) [127]. Another polymorphism of the FXIII-B subunit, His95Arg, was associated with mildly increased risk of venous thrombosis in Dutch Caucasians (OR 1.5, 95% CI 1.1–2.0) [128].

Other polymorphisms include the FXIII-A Tyr204Phe, which has been shown to be associated with a 9–11-fold increase in risk for MI and ischaemic stroke in a cohort of young Dutch women [129,130]; however, a similar association has not been demonstrated in a Brazilian population [131]. Mezei et al. [132] demonstrated that FXIII activity and FXIII-A_2_B_2_ antigen levels were significantly higher in females with VTE history compared to controls. FXIII-B levels were significantly lower in males with VTE than in the control group, highlighting an interesting sex-specific difference. Interestingly, neither p.His95Arg nor the intron K C > G polymorphism exerted any significant effect on the risk of VTE.

Inhibition of FXIIIa may be an avenue to achieve anticoagulation with limited bleeding risk with a few FXIIIa inhibitors currently under development, including small molecules and polypeptides. Of these, development of the hexapeptide-like ZED3197 and the polypeptide tridegin appear to be the most advanced. None are currently approved for use, and clinical trials are pending.

### 4.2. Physiological Role of Fibrinolysis

Fibrinolysis is the process of fibrin degradation that results in clot dissolution. The process involves the conversion of plasminogen to plasmin by tissue plasminogen activator (t-PA) or urokinase-type plasminogen activator (u-PA), which then degrades fibrin to fibrin degradation products, including D-dimer. The maintenance of an equilibrium between coagulation and fibrinolysis plays a vital role in the maintenance of homeostasis in order to prevent either uncontrolled thrombosis (defined as pathological formation of an intravascular clot in the absence of injury) or abnormal bleeding with potentially fatal consequences. If thrombus formation were to continue unchecked, it would lead to complete occlusion of the vessel in question and complete loss of blood flow downstream, resulting in catastrophic tissue damage, as is the case in acute MI, ischaemic stroke and acute peripheral vascular occlusion [133]. On the other hand, hyperfibrinolysis can lead to uncontrolled bleeding, as in the case of disseminated intravascular coagulation. Endogenous fibrinolysis (also known as spontaneous fibrinolysis) is therefore the body’s physiological countermeasure against lasting arterial or venous thrombosis.

The hallmark of the activation of the fibrinolytic system is the conversion of plasminogen into its active form, plasmin. Plasmin subsequently degrades insoluble fibrin fibers into soluble fibrin degradation products [134]. There are two physiological molecules that are capable of catalysing the conversion of plasminogen into plasmin. These are t-PA and urokinase-type plasminogen activator (u-PA) [135]. In the absence of fibrin, t-PA is a relatively poor plasminogen activator; however, its enzymatic activity is increased by orders of magnitude in the presence of fibrin, making it a fibrin-specific agent [27].

In healthy individuals, fibrinolytic activity regulation occurs at the level of plasminogen activation, mainly through the action of PAI-1 and PAI-2, which are effective against both t-PA and u-PA [134,136]. PAI-1 is the most abundant one and is produced by platelets and endothelial cells, whereas PAI-2 is produced by the placenta and is only detectable at appreciable levels during pregnancy [136,137].

Downregulation of the fibrinolytic system can also occur at the level of plasmin by the antagonizing effect exerted through A2AP [138]. A2AP forms a complex with plasmin, inhibits absorption of plasminogen onto fibrin and cross-links with FXIIIa to make fibrin more resistant to the effects of plasmin [133].

Another molecule capable of inhibiting the degradation of fibrin is TAFI [139]. It is activated following thrombin-mediated cleavage and acts by means of reducing plasminogen binding to partially degraded fibrin, as evidenced by increased LT [134,140]. The action of TAFI is greatly enhanced by thrombomodulin [141,142].

Finally, lipoprotein(a), Lp(a), is yet another modulator of fibrinolysis. Its molecular structure resembles that of plasminogen and it competitively binds to fibrin to exert its anti-fibrinolytic effect. In addition, it can also increase the rate of PAI-1 production by the endothelium, further reducing plasmin levels [143].

## 5. Measurement of Fibrinolysis

Although the physiological importance of endogenous fibrinolysis has been appreciated for decades, its measurement has been a challenge. There is no standard laboratory test to assess the fibrinolysis, and methods available are outlined below.

### 5.1. Assessment of Level/Activity of Individual Molecules (Factors) Regulating Fibrinolysis

As previously discussed, fibrinolysis involves a multitude of different molecules (plasmin, t-PA, urokinase, PAI-1/-2, A2AP, TAFI etc.) interacting with each other in a highly dynamic process. These individual molecules are produced in different locations, their individual concentrations may be augmented locally due to continuous flow of blood, and the magnitude of their respective effects in the fibrinolytic process as a whole can be challenging to accurately assess. As a result, measuring the level and/or the activity of individual molecules participating in the fibrinolysis pathway has so far been disappointing in producing prognostic data [144], with the exception of PAI-1, which has been shown to be a predictor of disease severity and all-cause mortality in sepsis [145]. This notion is exemplified by the assessment of t-PA. Studies have shown that high levels are in fact associated with greater cardiovascular risk, despite the physiological role of t-PA as potentiator of fibrinolysis [146,147].

### 5.2. Global Assessment of Fibrinolysis

Euglobulin clot lysis. This method, citrated plasma is acidified and incubated to form a precipitate to which (Ca^2+^) is then added to initiate clotting. The time taken to lyse the clot is measured. This is an outdated technique that has largely been replaced by newer methods described below.

#### 5.2.1. Plasma Clot Lysis Time (LT)

In this method, citrated platelet-poor plasma is obtained via centrifugation. The clotting process is initiated by the addition of (Ca^2+^) and a coagulation activator such as thrombin or recombinant tissue factor into the mixture. Use of recombinant tissue factor may produce more physiologically relevant results, since it takes into consideration the patients’ thrombin generation capacity [148]. Fibrinolysis is initiated by the addition of plasminogen activator. The mixture is then left static, and changes in turbidity are used to estimate thrombus formation and lysis. Fibrinolysis is defined as the time taken for the maximum turbidity to drop by 50%.

Both the above methods of fibrinolysis assessment require the external addition of activators of coagulation and fibrinolysis and are thus not accurate reflections of endogenous processes taking place in vivo. Another major limitation arises from the fact that they both fail to incorporate into their assessment the effect of any of the cellular components of whole blood. Finally, as they are both static tests, they fail to account for any effects that blood flow and shear stress (on platelets) have on both coagulation and fibrinolysis.

#### 5.2.2. Global Thrombosis Test (GTT) 

(Thromboquest Ltd., London, United Kingdom). This method measures coagulation and endogenous fibrinolysis in whole non-anticoagulated blood, most closely resembling physiological conditions in vivo. In this method, blood is subjected to flow under high shear stress, which results in platelet activation and aggregation. This leads to the arrest of blood flow due to the formation of an occlusive thrombus. The time taken to form an occlusive thrombus is termed occlusion time (OT) and is a marker of platelet reactivity and platelet thrombus formation. Due to subsequent endogenous fibrinolysis, the blood flow is eventually restored, following lysis of the initial occlusive thrombus. The time taken for the restart of flow is termed lysis time (LT) and is reflective of endogenous fibrinolysis activity. This method better mimics the conditions of clot formation (high shear stress), which are typically encountered in the arterial tree [144].

Viscoelastic methods include thromboelastography (TEG) (Haemoscope Corporation, Niles, Illinois) and the rotational thromboelastometry (ROTEM) (Tem International GmbH, Munich, Germany). Both tests provide information on clot formation, propagation, strength, stabilization and dissolution and are therefore able to provide a global assessment of coagulation and fibrinolysis. In TEG, whole or citrated blood can be used. The sample of blood is placed in a disposable cup, which undergoes constant rotational movement mimicking a low flow state similar to that encountered in the venous tree. Coagulation is activated by the addition of tissue factor or kaolin (for extrinsic and intrinsic pathways respectively). A pin is suspended in the blood sample, and as coagulation starts and clot formation is initiated, the varying forces exerted on the pin, as a result of viscoelasticity, are measured and plotted. Various indices are measured, including, but not limited to, the time (R) taken to form the initial clot and the clot strength (MA). Following that, the degree of clot lysis is measured at 30 and 60 min, reflecting fibrinolytic activity [144]. The main limitation of this technique is that it reflects static low flow thrombus formation, reflective of venous, rather than arterial, thrombosis. Furthermore, standard protocols (utilizing high tissue factor concentrations) are less sensitive in demonstrating clinically relevant hypofibrinolysis and are only sensitive to pronounced hyperfibrinolysis. Modified thromboelastometry protocols, with lower tissue factor concentrations and the addition of tissue plasminogen activator, may be of value [149,150].

## 6. Involvement of Fibrinolysis in Disease States

Patients presenting with ST-segment elevation myocardial infarction (STEMI) who achieve spontaneous reperfusion are believed to do so as a result of an effective endogenous fibrinolysis. This is associated with more favourable clinical outcomes, such as lower reinfarction rate, heart failure and overall lower mortality [151,152]. Impaired endogenous fibrinolysis in the context of acute coronary syndrome (ACS) has been shown to be associated with an increased rate of major cardiovascular adverse events (MACE) at 1 year and cardiovascular death. In a study of 300 patients presenting with ACS, impaired endogenous fibrinolysis, defined as LT in excess of 3000 sec, as assessed by GTT, occurred in some 20% of patients [153]. In a more recent study involving 496 patients presenting with STEMI, impaired endogenous fibrinolysis assessed upon arrival to the catheterisation laboratory was highly predictive of MI (HR 6.2, 95% CI 2.64–14.73; *p* < 0.001), MACE (HR: 9.1, 95% CI 5.29–15.75; *p* < 0.001) and cardiovascular death (HR 18.5, 95% CI 7.69–44.31; *p* < 0.001) at 1 year [154]. Importantly, the association remained significant following adjustment for baseline cardiovascular risk factors. The prognostic impact of impaired endogenous fibrinolysis was also assessed in a sub-study of the PLATO study by Sumaya et al. [155]. This was performed by obtaining blood samples on discharge following hospitalisation for ACS in 4354 patients. Assessment of fibrinolysis potential, as measured by clot LT (defined as time to achieve 50% reduction in sample turbidity), correlated with cardiovascular death and MI at 1 year, following adjustment for known cardiovascular risk factors (HR 1.17, 95% CI 1.05–1.31; *p* < 0.01).

Similar to patients with ACS, differences in endogenous fibrinolysis have also been observed in acute ischaemic stroke populations. In a study of 335 individuals, out of which 103 had an ischaemic stroke, clot LT was longer in patients than that in controls [60]. A study of 74 patients with ischaemic stroke, short CLT on admission to hospital was predictive of favorable outcome at 3 months [156]. Similarly, Drabik et al. [157] showed that CLT—in addition to the CHA_2_DS_2_-VASc score—was predictive of future events over a median follow up period of 4.3 years in 236 patients with atrial fibrillation who were treated with vitamin K antagonists. Taomoto et al. [158] compared 185 patients with acute cerebral infarction with 195 healthy volunteers, two weeks following the index event. They showed that the endogenous fibrinolytic activity in patients was significantly impaired (longer LT) compared to healthy individuals (3159 ± 1549 s vs. 2231 ± 1223 s respectively), despite antiplatelet medication. Prior to antiplatelet therapy initiation, the LT of patients was prolonged even further.

The thrombotic and thrombolytic status of 216 patients with ESRD on hemodialysis was assessed by Sharma et al. [159] using the GTT. Impaired endogenous fibrinolysis (defined as LT > 3000 sec) was strongly associated with MACE (HR 4.25, 95% CI 1.58–11.46, *p* = 0.004), non-fatal MI and stroke (HR 14.28, 95% CI 1.86–109.90, *p* = 0.01), and peripheral thrombosis (HR 9.08, 95% CI 2.08–39.75, *p* = 0.003). However, no association was found between OT and MACE. Finally, LT has been shown to be significantly prolonged in male habitual smokers compared to non-smokers by Suehiro et al. [160], suggesting attenuation of spontaneous fibrinolytic activity due to smoking.

Two studies utilising viscoelastic methods in the assessment of endogenous fibrinolysis in stroke have been unable to demonstrate any clinically relevant differences. ROTEM was utilised in a study of 143 individuals with stroke [161] but did not reveal any significant differences in coagulation and fibrinolysis parameters between patients and controls or to associate fibrinolytic activity to stroke severity. In a study of 171 patients with ischaemic stroke, before and after t-PA administration, TEG was unsuccessful in detecting responders from non-responders to t-PA therapy [162]. These results might partially be attributed to the limitations of standard viscoelastic protocols in detecting clinically relevant changes of fibrinolytic capacity, as discussed at the end of the previous section.

Assessment of fibrinolytic factors in patients with stroke does appear to relate to clinical outcomes. In 109 patients with ischaemic stroke, higher TAFI levels on admission were associated with more severe National Institutes of Health Stroke scale and disability in patients receiving or not receiving thrombolysis [163]. In another small study of 43 patients with acute ischaemic stroke treated with thrombolysis, an association was found between higher PAI-1 levels on admission and angiographic failure of culprit cerebral artery recanalisation [164].

Studies assessing fibrinolytic potential in venous thrombosis [66,165,166] have associated prolonged clot LT with a two-fold increase in the prevalence of thrombotic events. Hypofibrinolysis, as assessed by a plasma-based clot lysis assay, has been associated with increased risk of a first venous or arterial event but not with recurrence of venous thrombosis [167]. In addition, a study of 704 patients who had experienced unprovoked VTE reported an association between clot LT (split into quartiles) and risk of future VTE recurrence in women [168].

## 7. Conclusions

The equilibrium between coagulant and fibrinolytic pathways plays an important role in regulating thrombosis and hemostasis. There is growing evidence to suggest that mechanical and biochemical properties of the fibrin clot, the fibrinolysis-inhibitory effect of FXIII and overall impairment of endogenous fibrinolysis all play a crucial role in the dynamic process of clot formation and dissolution and contribute significantly to the pathophysiology of cardiovascular events. Further studies are needed with clinically relevant point-of-care global assays to assess the fibrinolytic function and the potential to improve this to reduce cardiovascular risk.

## Abbreviations

A2AP	alpha 2-antiplasmin
ACS	acute coronary syndrome
CAD	coronary artery disease
FXIII/FXIIIa	coagulation factor XIII/activated coagulation factor XIII
GTT	Global Thrombosis Test
MACE	major cardiovascular adverse events
MI	myocardial infarction
PAI-1/-2	Plasminogen activator inhibitor-1/-2
ROTEM	Thromboelastometry
TAFI	Thrombin activatable fibrinolysis inhibitor
TEG	Thromboelastography
VTE	venous thromboembolism
